# Analysis of continuous first-line treatment with docetaxel and carboplatin for advanced non-small cell lung cancer

**DOI:** 10.3892/ol.2014.1973

**Published:** 2014-03-14

**Authors:** TAKUYA AOKI, AKINORI EBIHARA, YURIKA YOGO, KEIICHI SUEMASU, FUMIO SAKAMAKI

**Affiliations:** 1 Division of Respiratory Medicine, Department of Internal Medicine, Tokai University School of Medicine, Isehara, Kanagawa 259-1193, Japan; 2 Division of Respiratory Medicine, Department of Internal Medicine, Saiseikai Central Hospital, Minato-ku, Tokyo 108-0073, Japan; 3 Department of Thoracic Surgery, Saiseikai Central Hospital, Minato-ku, Tokyo 108-0073, Japan

**Keywords:** continuous first-line treatment, docetaxel, carboplatin, efficacy, safety

## Abstract

The present study aimed to analyze the efficacy and safety of multiple cycles of docetaxel and carboplatin (CBDCA) as a first-line treatment in patients with advanced non-small cell lung cancer (NSCLC). Patients with stage III or IV NSCLC, whose treatment began between July 1999 and February 2003, were retrospectively evaluated. Relatively low doses of docetaxel and CBDCA were administered for as many cycles as possible. The primary outcome assessed was the overall survival (OS) time, and the secondary outcomes included progression-free survival (PFS) time, response rate (RR) and adverse events. The median cycle number was four (range, 2–12). The median OS time was 400 days, and for adenocarcinoma and non-adenocarcinoma, the OS time was 490 and 192 days, respectively. The median PFS time was 176 days and the RR was 66.7%. The main toxicity of the treatment was neutropenia, with grade 3 or 4 neutropenia occurring in 81.0% of patients. Continuous first-line treatment with this regimen may have encouraging effects within a certain group of advanced NSCLC patients, thereby warranting further investigations.

## Introduction

For almost 20 years lung cancer has been the most frequent malignancy worldwide, and it remains the most common cause of cancer mortality (1,2). A large amount of evidence supports the use of chemotherapy as a first-line therapy in advanced non-small cell lung cancer (NSCLC) patients with a good performance status (PS). This is founded on a landmark meta-analysis that demonstrated the reduced risks of mortality and an increased one-year survival rate following chemotherapy (3). The current standard first-line therapy consists of platinum combinations of two cytotoxic drugs with or without a molecular-targeted agent (4,5).

The optimal duration of first-line chemotherapy for patients with stage IV NSCLC has yet to be established. Socinski *et al* (6) demonstrated that continuing treatment with carboplatin (CBDCA) and paclitaxel beyond four cycles produced no overall benefit in survival time, response rates or quality of life for patients with advanced NSCLC. However, the data are limited. According to the 2011 focused update of the 2009 American Society of Clinical Oncology (ASCO) clinical practice guideline (7), first-line cytotoxic chemotherapy should be discontinued when disease progression has been detected or following four cycles in patients whose disease is stable but is not responding to treatment. For the patients with stable disease (SD), or for those who show a response following four cycles of treatment, immediate treatment with an alternative, single-agent chemotherapy, including pemetrexed, docetaxel or erlotinib, may be administered. Following a fixed course of treatment, a break from cytotoxic chemotherapy is also acceptable, however, second-line chemotherapy is initiated upon disease progression.

Despite the development of various first-line therapies with a fixed course of treatment, the outcomes remain poor. Two varying types of chemotherapy for the continuation of treatment soon after stopping the platinum-based doublet regimens have been extensively investigated. One is continuation maintenance therapy, which involves continuing an agent that was part of the initial induction treatment regimen (8–10), while the other is switch maintenance therapy that involves initiating another agent prior to disease progression (10–15). The approximate median progression-free survival (PFS) time with these therapeutic approaches ranged between 3 and 5 months following maintenance therapy initiation, and between 6 and 7 months following induction therapy initiation, although patients showing progressive disease (PD) with the first-induction therapy were excluded (8–15).

One of the major variations between first-line platinum doublet combinations and maintenance therapies is feasibility. Continuation of a first-line platinum doublet regimen may be difficult, even when the regimen is effective, due to toxicity. Feasible first-line platinum doublet combinations could be continued beyond standard cycle numbers and may exert favorable effects if cumulative toxicities do not occur or can be managed without deterioration of the PS. It is, however, extremely difficult to perform a prospective study on continuous multiple-cycle first-line treatments under the present circumstances. However, the cycle number of first-line treatment was not strictly determined until 2003 (16). The present study retrospectively analyzed the efficacy and safety of continuous multiple-cycle first-line treatment with CBDCA and docetaxel in advanced NSCLC patients whose treatment had been initiated during the period between July 1999 and February 2003.

## Materials and methods

### Patient selection

Between July 1999 and February 2003, NSCLC patients who met the established criteria were treated with docetaxel plus CBDCA at Saiseikai Central Hospital (Tokyo, Japan), according to the selected treatment schedule. In total, a case series of 21 patients (17 males and 4 females) with inoperable stage IIIB and IV NSCLC without prior chemotherapy were evaluated. The medical data collected up to July 2004 was assessed. The primary outcome was overall survival (OS) time, and the secondary outcomes included PFS, response rate (RR) and adverse events. The retrospective protocol was approved by the institutional review boards of the Tokai University School of Medicine (Isehara, Japan) and the Saiseikai Central Hospital (Tokyo, Japan).

The selection criteria for the patients were as follows: Histologically or cytologically proven NSCLC; an Eastern Cooperative Oncology Group PS of 0, 1 or 2; an age of ≥18 years; measurable lesions as assessed by computed tomography (CT); adequate bone marrow reserves, defined as a white blood cell (WBC) count of ≥3,500/μl, a neutrophil count of ≥2,000/μl, a platelet count of ≥100,000/μl and hemoglobin levels that were ≥10 g/dl; and adequate liver function, defined as bilirubin levels of <2.0 mg/dl and/or aspartate transaminase (AST), alanine transaminase (ALT) and γ-glutamyltransferase levels at more than three times the upper limit of normal. Patients with impaired renal function were included if the measured creatinine clearance was >40 ml/min. Patients with ischemic heart diseases and diabetes mellitus were included if their conditions were medically controlled, and those who had central nervous system metastasis were also included. None of the patients had undergone previous radiation therapy or major surgery. Patients with malignant pleural and/or pericardial effusions were also included. Patients with stage IIIB disease who could be treated with curative radiotherapy were excluded.

### Treatment schedule

Prior to starting chemotherapy, all the patients underwent a physical examination, a complete blood count, blood and urine chemistry, a chest X-ray, a chest CT scan, an abdominal echo and/or CT scan, a head CT and/or MRI, a bone scan and electrocardiography. The creatinine clearance was measured, and complete blood cell counts, differential counts and routine blood chemistry measurements were performed at least twice weekly during the first cycle. On the day of the nadir following the initiation of chemotherapy, the minimum WBC and neutrophil and platelet counts were determined. The patients were hospitalized during all the chemotherapy courses and evaluated by physical examination, complete blood count, blood chemistry and a chest X-ray. The responses were assessed every two cycles by a chest X-ray and/or a CT scan, and metastasis was also evaluated.

All the patients received the following treatment protocol: 60 mg/m^2^ docetaxel as a 3-h infusion in 500 ml of 5% glucose or 0.9% saline, followed by 300 mg/m^2^ CBDCA as a 2-h infusion in 500 ml of 5% glucose or 0.9% saline solution on day 1. Antiemetic treatment with 5-hydroxytryptamine-3 antagonists was provided prior to the administration of chemotherapy. Steroids were not administered as premedication. The treatment was repeated every three to four weeks, provided that the patients had sufficiently recovered from any toxicities. The patients were treated with the same regimen as many times as possible unless there was evidence of PD, unacceptable toxicity or they refused further treatment. Once PD occurred following the initiation of the first-line treatment, second-line treatment began if the patient wanted to continue. The patients with a brain metastasis received either γ-knife radiosurgery or whole-brain radiotherapy. The patients with symptomatic bone metastasis and invasion were also treated with radiotherapy.

Recombinant human granulocyte colony-stimulating factor (G-CSF) was not administered prophylactically in the first cycle. G-CSF was administered at a dose of 1 μg/kg when the neutrophil counts were <500/mm^3^, the febrile neutrophil counts were <1,000/mm^3^ or the leukocyte counts were <2,000/mm^3^. The patients who experienced grade 4 neutropenia received prophylactic G-CSF administration and the CBDCA dose was reduced by 25% in subsequent courses. The docetaxel dose was reduced by another 25% to ameliorate the neutropenia, as necessary. If the measured creatinine clearance was between 40 and 50 ml/min, the CBDCA dose was reduced by 25%.

### Response and toxicity evaluation

The responses were assessed using Response Evaluation Criteria in Solid Tumors (version 1.0) (17). The confirmation of a complete response (CR) or partial response (PR) was required at least 4 weeks subsequent to the initial documentation. SD was defined as disease control (absence of progression) when it was maintained for at least 6 weeks. Toxicity was graded according to the National Cancer Institute’s Common Toxicity Criteria for Adverse Events, version 3.0 (18).

### Statistical analysis

PFS time was defined as the time that elapsed between the first day of the first-line therapy and the date of PD or mortality. The OS time was defined as the elapsed time between the first day of the first-line therapy and the date of mortality. The PFS and OS time differences between the groups were analyzed using the Kaplan-Meier method and compared using the log-rank test. Differences were considered to indicate a statistical significance when P<0.05.

## Results

The patient characteristics are listed in Table I. Unspecified NSCLC included poorly-differentiated and non-specified carcinomas. All the patients had a PS of 0–2. The measured creatinine clearances were 40–50, 51–60, 61–80, 81–90 and ≥91 ml/min in three (14.3%), four (19.0%), four (19.0%), five (23.8%) and five (23.8%) patients, respectively. The median area under the plasma concentration vs. time curve (AUC) as calculated by employing Calvert’s formula, with the measured creatinine clearance as the glomerular filtration rate, was 4.4 mg/ml × min (range, 2.8–6.1 mg/ml × min). The calculated AUC (mg/ml × min) values were 2.5–3.0, 3.1–4.0, 4.1–4.5, 4.6–5.0, 5.1–6.0 and ≥6.1 in two (9.5%), four (19.0%), five (23.8%), five (23.8%), four (19.0%) and one (4.8%) patient, respectively. The vast majority of patients presented with adenocarcinoma and stage IV disease. All the patients were assessable for toxicity and response.

Fourteen patients achieved a PR (Table II). The overall RR was 66.7% [95% confidence interval (CI), 43.0–85.4]. In total, five patients (23.8%) had SD and two (9.5%) had PD. The patients with adenocarcinoma had an RR of 64.3% (95% CI, 35.1–87.2), and those with non-adenocarcinoma had an RR of 71.4% (95% CI, 29.0–96.3). The median number of cycles per patient was four (range, 2–12 cycles), and four patients received ≥10 cycles and achieved long survival durations; 823 days (11 cycles), 625 days (10 cycles), 708 days (12 cycles) and 496 days (10 cycles).

**Figure 1:**
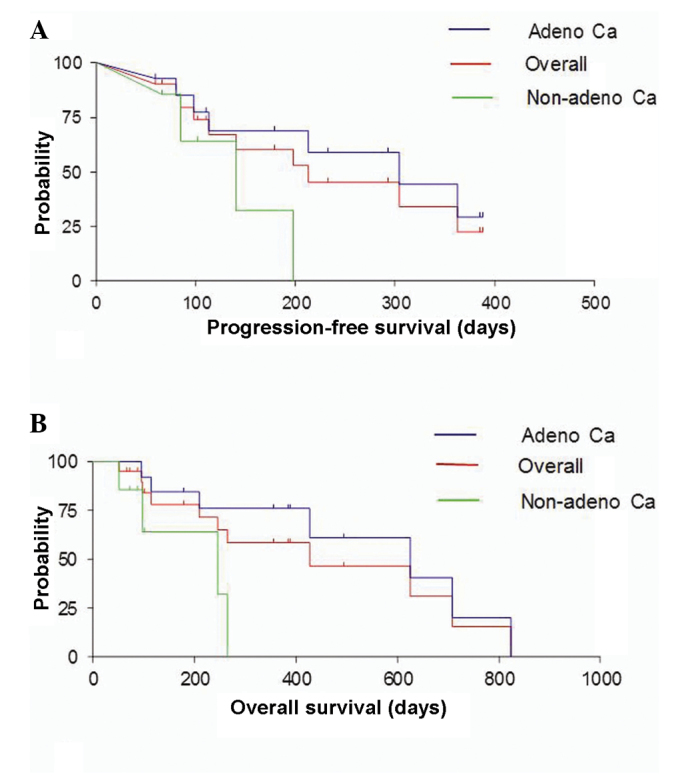
Cumulative Kaplan-Meier curves for (A) PFS and (B) OS, stratified according to tumor histology. Ca, carcinoma; PFS, progression-free survival; OS, overall survival.

The median OS time (Fig. 1) was 400 days (range, 52–1,047 days). For the adenocarcinoma patients the overall median survival time was 490 days (range, 95–1,047 days) and for the non-adenocarcinoma patients it was 192 days (range, 52–297 days). The log-rank test demonstrated a significant survival difference between the adenocarcinoma and non-adenocarcinoma patients (P=0.0012). The one-year survival rate was 47.6% (95% CI, 25.7–70.2) and the two-year survival rate was 9.5% (95% CI, 1.2–30.4). Among the adenocarcinoma patients, the one-year survival rate was 71.4% (95% CI, 41.9–91.6) and the two-year survival rate was 14.3% (95% CI, 1.8–42.8). The one-year survival rate was, however, 0% (95% CI, 0–41.0) among the non-adenocarcinoma patients. The median PFS time (Fig. 1) for all the NSCLC patients was 176 days (range, 31–388 days), while the median PFS time was 210 days (range, 42–388 days) for the adenocarcinoma patients and 108 days (range, 31–198 days) for the non-adenocarcinoma patients.

The actual and relative dose intensities of CBDCA are shown in Table III. The cycle number and the total number of G-CSF injections for each patient are also shown. Dose reduction was necessary in six patients. Neutropenia was the only toxicity for which dose reduction was required (Table IV). No patient experienced a dose reduction for any reason other than grade 4 neutropenia. Grade 3/4 neutropenia was observed in 17 patients (81.0%), grade 3/4 leukocytopenia in 14 (66.7%), grade 3 thrombocytopenia in three (14.3%) and grade 3 anemia in one patient (4.8%). Neither grade 4 thrombocytopenia nor grade 4 anemia occurred. Febrile neutropenia was observed in four patients. All these patients were successfully managed with G-CSF and broad-spectrum antibiotics. The patients with neutropenia recovered with G-CSF administration, however, one patient with a perirectal abscess deteriorated while neutropenic. This infection was successfully treated with broad-spectrum antibiotics. No thrombopenic episodes were complicated by hemorrhage. Alopecia was almost universal (95.2%), and grade 1/2 nausea was observed in 10 patients (47.6%), whereas grade 1/2 vomiting was observed in three (14.3%). Grade 1/2 diarrhea occurred in four patients (19.0%) and a grade 1 allergic reaction in one (4.8%). Neuropathy, i.e., paresthesia with a loss of vibration sensation, occurred in only one patient. None of the patients developed pneumonitis or interstitial pneumonia. Grade 1/2 elevations of hepatic enzymes were observed in four (AST), four (ALT) and two (alkaline phosphatase) patients and these levels normalized without medication. None of the patients showed nephrotoxicity and there were no treatment-related mortalities.

## Discussion

The present study retrospectively analyzed the continuation of first-line treatment with docetaxel and CBDCA in advanced NSCLC patients. The continuation of this first-line treatment for disease control was possible, as the only cumulative toxicity was neutropenia. The median number of cycles was, however, four (range, 2–12 cycles), the same as the ASCO recommendations (7). This continuous combination therapy may have encouraging activity for the treatment of stage IV adenocarcinoma, although not for stage IV non-adenocarcinoma, including the poorly-differentiated type. In the treatment of the adenocarcinoma patients of the present study, the RR of 64.3%, the median PFS time of 210 days, the median survival time of 490 days and the one-year survival rate of 71.4% were encouraging. Among the adenocarcinoma patients who received ≥10 cycles, long-term survival durations were achieved. Despite tumor persistence, neither tumor regrowth nor progression occurred until the terminal stage in these patients. The feasibility of the CBDCA-based combination with docetaxel makes multiple administrations possible without serious side-effects, including nausea/vomiting and nephrotoxicity, which often develop in patients receiving CDDP-based combinations, or the cumulative muscle pain and neurotoxicity observed in those who receive paclitaxel.

Regarding the cycle number for first-line treatment, a few studies have confirmed the non-inferiority, in terms of the OS time, of three to four cycles compared with six cycles of chemotherapy (19,20). However, a study by Soon *et al* (21) reported that extending chemotherapy beyond a standard number of cycles significantly improved the PFS time in a meta-analysis of 13 randomized-controlled trials involving >3,000 patients. A cycle number exceeding six could be administered in certain patients, particularly those with adenocarcinoma, according to individual PS and the side-effects of this protocol. The profiles of toxicities occurring in the first cycle were almost the same as those in the subsequent cycles. Toxicities, which had not been observed in the first several cycles, were not cumulative.

Limitations of the present retrospective results, obtained in clinical settings, include the small number of patients and the lack of epidermal growth factor receptor mutation status and echinoderm microtubule-associated protein-like 4 anaplastic lymphoma kinase fusion gene status. However, determination of these genetic features was not possible between 1999 and 2003. With this treatment protocol, as performed at Saiseikai Central Hospital (Tokyo, Japan) prior to 2003, the CBDCA dose was determined based on the body surface area (BSA), rather than by the AUC, and the CBDCA dose was 300 mg/m^2^, which is the dose approved by the Japanese Ministry of Health, Labour and Welfare. Therefore, Calvert’s formula was used to calculate the corresponding AUC, employing the measured creatinine clearance. The mean calculated AUC was 4.4 mg/ml × min, which was low compared with the TAX 326 study, a phase III study of docetaxel combined with a platinum agent (22). In the TAX 326 study, the AUC was 6 mg/ml × min for CBDCA, and the docetaxel dose was 75 mg/m^2^.

Neutropenia was a serious issue, and numerous G-CSF administrations were necessary in patients receiving this continuation therapy of docetaxel and CBDCA. No other toxicities were serious. Despite the relatively low administered doses of docetaxel and CBDCA, 17 patients (81.0%) experienced grade 3/4 neutropenia. These patients were successfully managed with G-CSF and a dose reduction. No grade 3/4 toxicities, other than myelosuppression, were observed. Alopecia was almost universal, but not serious. Nausea, vomiting and diarrhea were moderate, but manageable. There were no serious allergic reactions. Neuropathy, which often occurs with paclitaxel administration, was rare and mild. Due to these tolerable toxicity profiles and as hydration was not necessary, patients with renal impairment (creatinine clearance, 40–50 ml/min) were successfully treated with this regimen.

Continuous first-line chemotherapy with docetaxel (60 mg/m^2^) and CBDCA (300 mg/m^2^), as determined by BSA rather than AUC, may be effective in advanced NSCLC patients with adenocarcinoma. In the present study, the doses of docetaxel and CBDCA were set relatively low, compared with the majority of previously reported studies, and administered as many times as possible. The rate of neutropenia was high however, possibly making prophylactic G-CSF administration necessary. Further investigations of the optimal cycle numbers are warranted.

## Figures and Tables

**Table I:** Patient characteristics.

Characteristics	Value (%)
Total patients, n	21 (100)
Gender, n	
Male	17 (81.0)
Female	4 (19.0)
Age, years	
Median	65
Range	46–77
ECOG PS, n	
0	10 (47.6)
1	4 (19.0)
2	7 (33.3)
Creatinine clearance in ml/min, n	
40–50	3 (14.3)
51–60	4 (19.0)
61–80	4 (19.0)
81–90	5 (23.8)
≥91	5 (23.8)
AUC^a^ in mg/ml × min, n	
2.5–3.0	2 (9.5)
3.1–4.0	4 (19.0)
4.1–4.5	5 (23.8)
4.6–5.0	5 (23.8)
5.1–6.0	4 (19.0)
≥6.1	1 (4.8)
Histology, n	
Adenocarcinoma	14 (66.7)
Squamous cell carcinoma	3 (14.3)
Large-cell carcinoma	2 (9.5)
Unspecified NSCLC	2 (9.5)
Clinical stage, n	
IIIB	5 (23.8)
IV	16 (76.2)
No. of organs with metastases, n	
0 (stage IIIB)	5 (23.8)
1	3 (14.3)
2	6 (28.6)
≥3	7 (33.3)

a AUC, vs. time curve, calculated by Calvert’s formula.

ECOG, Eastern Cooperative Oncology Group; AUC, area under curve; NSCLC, non-small cell lung cancer; PS, performance status.

**Table II:** Responses according to patient characteristics.

		Response, n					
				
Characteristics	n	CR	PR	SD	PD	CR+PR,	% 95% CI
Histological subtypes, Ca							
Adeno	14	0	9	4	1	64.3	35.1–87.2
Non-adeno	7	0	5	1	1	71.4	29.0–96.3
Squamous cell	3	0	3	0	0		
Large cell/unspecified	4	0	2	1	1		
Overall	21	0	14	5	2	66.7	43.0–85.4
No. of chemotherapy cycles							
2	5	0	3	0	2		
3	3	0	0	3	0		
4	5	0	4	1	0		
6	1	0	1	0	0		
7	3	0	3	0	0		
≥10	4	0	3	1	0		

Ca, carcinoma; CR, complete response; PR, partial response; SD, stable disease; PD, progressive disease; CI, confidence interval.

**Table III:** Actual and relative CBDCA doses.

CBDCA dose, mg/body	Cycle no.	G-CSF^a^ vials	Dose intensity of CBDCA, cycles										

			1	2	3	4	5	6	7	8	9	10	>10
300	4	0	1	1	1	1							
360	7	0	1	1	1	1	1	1	1				
400	2	16	1	1									
400	3	11	1	1	1								
400	4	12	1	1	1	1							
420	2	7	1	0.75									
420	3	0	1	1	1								
450	4	12	1	1	1	1							
450	7	8	1	1	1	1	0.75	0.75	0.75				
450	10	39	1	1	0.75	0.75	0.75	0.75	0.75	0.75	0.75	0.75	
450	7	12	1	1	1	0.75	0.75	0.75	0.75				
450	10	21	1	1	1	1	1	1	1	1	1	1	
450	11	32	1	1	1	1	1	1	1	1	1	1	1
450	12	53	1	1	1	1	1	1	1	1	1	0.75	0.75
460	6	34	1	0.75	0.75	0.75	0.75	0.75					
480	2	14	1	1									
500	2	9	1	1									
500	2	0	1	1									
500	4	6	1	1	1	1							
550	3	0	1	1	1								
585	4	7	1	1	1	1							

a 50 μg G-CSF/vial.

CBDCA, carboplatin; G-CSF, granulocyte colony-stimulating factor.

**Table IV:** Toxicity in patients with National Cancer Institute’s Common Toxicity Criteria for Adverse Events (most severe, any course).

Toxicity	Grade				Total, n (%)	≥Grade 3, n (%)

	1	2	3	4		
Neutropenia	0	2	7	10	19 (90.5)	17 (81.0)
Leukocytopenia	2	2	11	3	18 (85.7)	14 (66.7)
Febrile neutropenia	0	0	4	0	4 (19.0)	4 (19.0)
Thrombocytopenia	4	1	3	0	8 (38.1)	3 (14.3)
Infection	0	0	1	0	1 (4.8)	1 (4.8)
Anemia	6	7	1	0	14 (66.7)	1 (4.8)
Alopecia	9	11	0	0	20 (95.2)	0 (0.0)
Nausea	8	2	0	0	10 (47.6)	0 (0.0)
Vomiting	2	1	0	0	3 (14.3)	0 (0.0)
Diarrhea	2	2	0	0	4 (19.0)	0 (0.0)
Ileus	0	0	0	0	0 (0.0)	0 (0.0)
Allergic reaction	1	0	0	0	1 (4.8)	0 (0.0)
Neuropathy	1	0	0	0	1 (4.8)	0 (0.0)
Pneumonitis	0	0	0	0	0 (0.0)	0 (0.0)
AST	1	3	0	0	4 (19.0)	0 (0.0)
ALT	1	3	0	0	4 (19.0)	0 (0.0)
Alkaline phosphatase	1	1	0	0	2 (9.5)	0 (0.0)
Bilirubin	0	0	0	0	0 (0.0)	0 (0.0)
Liver failure	0	0	0	0	0 (0.0)	0 (0.0)

AST, aspartate transaminase; ALT, alanine transaminase.
